# The hard part we often forget: providing care to children and adolescents with advanced HIV disease

**DOI:** 10.1002/jia2.26041

**Published:** 2023-03-21

**Authors:** Lisa Jane Frigati, Diana Gibb, Joseph Harwell, Judith Kose, Victor Musiime, Helena Rabie, Ajay Rangaraj, Pablo Rojo, Anna Turkova, Martina Penazzato

**Affiliations:** ^1^ Department of Paediatrics and Child Health Stellenbosch University, Tygerberg Academic Hospital Cape Town South Africa; ^2^ Medical Research Council Clinical Trials Unit at University College London London UK; ^3^ Clinton Health Access Initiative Boston Massachusetts USA; ^4^ Technical Strategy and Innovation The Elizabeth Glaser Pediatric AIDS Foundation Nairobi Kenya; ^5^ Erasmus MC Department of Viroscience Erasmus University Rotterdam Netherlands; ^6^ Department of Paediatrics and Child Health Makerere University Kampala Uganda; ^7^ Research Department Joint Clinical Research Centre Kampala Uganda; ^8^ World Health Organization Geneva Switzerland; ^9^ Department of Pediatrics Hospital Universitario Doce de Octubre Madrid Spain

**Keywords:** adolescents, advanced HIV disease, children, co‐infections, prevention, treatment

## Abstract

**Introduction:**

Many children and adolescents living with HIV still present with severe immunosuppression with morbidity and mortality remaining high in those starting antiretroviral therapy (ART) when hospitalized.

**Discussion:**

The major causes of morbidity and mortality in children living with HIV are pneumonia, tuberculosis, bloodstream infections, diarrhoeal disease and severe acute malnutrition. In contrast to adults, cryptococcal meningitis is rare in children under 5 years of age but increases in adolescence. In 2021, the World Health Organizations (WHO) consolidated guidelines for managing HIV disease and rapid ART included recommendations for children and adolescents. In addition, a WHO technical brief released in 2020 highlighted the various interventions that are specifically related to children and adolescents with advanced HIV disease (AHD). We discuss the common clinical presentations of children and adolescents with AHD with a focus on diagnosis, prevention and treatment, highlight some of the challenges in the implementation of the existing package of care, and emphasize the importance of additional research to address the needs of children and adolescents with AHD.

**Conclusions:**

There are limited data informing these recommendations and an urgent need for further research on how to implement optimal strategies to ensure tailored approaches to prevent and treat AHD in children and adolescents. Holistic care that goes beyond a simple choice of ART regimen should be provided to all children and adolescents with AHD.

## 1 INTRODUCTION

Around 30% of children and adolescents living with HIV still present with severe immunosuppression at HIV diagnosis [1]. Mortality is high in children starting antiretroviral therapy (ART) who are hospitalized [2]. The major causes of morbidity and mortality among children living with HIV (CLHIV) in low‐ and middle‐income countries are pneumonia, tuberculosis (TB), bloodstream infections, diarrhoeal disease and severe acute malnutrition (SAM) [3].

The clinical presentation of “advanced HIV disease” (AHD) is different in children compared to adolescents and adults. The causes of morbidity can differ as the child progresses from the neonatal period through infancy, childhood and finally adolescence. For example, while cryptococcal meningitis is a major driver of morbidity and mortality in adults with AHD, it is rare in children between the ages of 1 and 5 years, with peaks occurring in infancy and adolescence [4, 5]. These differences in the prevalence of certain opportunistic infections and clinical presentations are essential to inform appropriate approaches to AHD despite the lack of paediatric‐specific data in this population.

The 2021 World Health Organizations (WHO) consolidated guidelines for managing HIV, along with a WHO technical brief from 2020, recommend a package of care specifically tailored to children and adolescents [6, 7]. The main interventions known to reduce morbidity and mortality among CLHIV are screening for TB and malnutrition, treatment of TB, pneumocystis pneumonia (PJP), severe pneumonia and malnutrition and prevention of PJP, TB and other common vaccine‐preventable infections. Screening and pre‐emptive cryptococcal therapy is an important strategy for adolescents. These evidence‐based strategies were summarized as **S**creen (for TB, malnutrition, cryptococcal meningitis), **T**reat (TB, PJP, pneumonia, severe bacterial infections), **O**ptimize (early ART start and adherence counselling) and **P**revent (TB, PJP, cryptococcal meningitis, severe bacterial infections with vaccines) (**STOP**‐AIDS) [6].

According to WHO, children less than 5 years of age should all be considered as having advanced HIV regardless of CD4 count or WHO Staging. Children older than 5 years and adolescents with a WHO HIV Stage 3 or 4 or a CD4 cell count <200 cells/mm^3^ are defined as having AHD. These definitions refer to children who are newly diagnosed or are yet to initiate ART but children and adolescents who have had a period of ART interruption and are now re‐engaging with care should be assessed for AHD and should be offered the package of care as appropriate. Access to CD4 cell count testing remains of great importance to define AHD and to inform the initiation and discontinuation of various prevention interventions, such as cotrimoxazole prophylaxis.

We aim to highlight the clinical presentations of AHD in this population, as well as some of the challenges in the implementation of the existing package of care, and the need for additional research to address the needs of children and adolescents with AHD.

## 2 DISCUSSION

### 2.1 Common conditions in children and adolescents with AHD

#### 2.1.1 Tuberculosis

TB remains one of the most common causes of morbidity in CLHIV despite the widespread use of ART. WHO guidelines recommend systematic screening for TB disease in CLHIV less than 10 years old using a symptom screen, including current cough, fever, poor weight gain or close contact with a TB patient [8, 9]. Adolescents should be screened for TB disease using the adult four‐symptom screen (current cough, fever, weight loss or night sweats), and those who report any one of the four symptoms should be evaluated further using molecular testing if available [8, 9].

In a study that assessed the capacity to diagnose TB in children in sub‐Saharan Africa (SSA), of 651 sites surveyed, only 39 (6%) had the capacity to perform induced sputa and 5% could collect gastric aspirates [10]. WHO has now endorsed the use of lateral flow urine lipoarabinomannan assay as well as stool and nasopharyngeal aspirates as additional samples that can be used for molecular testing to diagnose TB in children [9].

Despite technological advances, many children with TB are still diagnosed clinically and may present with chronic cough, fever, lymphadenopathy, failure to thrive, signs of acute pneumonia, chronic diarrhoea, decrease in playfulness, listlessness or signs of meningitis.

Clinical scoring systems such as the one developed and internally validated in the PAANTHER study could be used to diagnose symptomatic children with TB. The score, used to identify pulmonary TB in CLHIV < 14 years of age, was employed in four countries in SSA and Southeast Asia and integrated clinical features, chest radiographs, abdominal ultrasound and interferon‐gamma release assays (IGRAs).

The optimal scoring tool (incorporating IGRA) had a sensitivity of 89% and a specificity of 61% for diagnosing pulmonary TB [11].

This tool can be adapted to different settings with varying resources and may enable the timely initiation of TB treatment in CLHIV. Compared with microbiologic assays, the decision tool may improve diagnostic sensitivity by 60% [13].

Paediatric‐friendly, fixed‐dose combinations to treat TB and updated weight‐based dosing charts are now available. Recently, a phase 3 randomized clinical trial in 1204 children with minimal pulmonary disease, that included 127 CLHIV, showed that 4 months of TB treatment (2 months of isoniazid (H), rifampicin (R), pyrazinamide (Z) and ethambutol (E), (2HRZE) followed by 2 months of (HR)) were as effective as 6 months of treatment [14]. This is now recommended by the WHO for children and adolescents between 3 months and 16 years of age with a minimal disease in whom there is no suspicion of drug‐resistant TB [9].

In adolescents 12 years or older living with HIV, with CD4 counts greater than 100 cells/ml^3^, a treatment‐shortening regimen with 2 months of H, rifapentine, moxifloxacin and pyrazinamide, followed by 2 months of H, rifapentine moxifloxacin was non‐inferior to a standard 6 months regimen of 2HRZE/4HR [15]. Data on how these regimens may be implemented in high‐prevalence HIV countries are needed.

#### 2.1.2 Pneumonia

Establishing an aetiological diagnosis of pneumonia in CLHIV is difficult due to overlapping clinical features, limited availability of diagnostic tests and challenges in the interpretation of microbiological isolates of respiratory specimens due to high rates of polymicrobial infections and colonization. The most common bacterial pathogens causing pneumonia in CLHIV are streptococcus (predominantly *S pneumoniae*) and *Staphylococcus aureus*. These pathogens can be effectively treated with commonly available antibiotics, such as amoxicillin, cloxacillin and amoxicillin/clavulanic acid [9, 15]. However, infections in CLHIV are often polymicrobial (including viruses, fungi and Gram‐negative bacteria) and this should be taken into account when treating empirically [16].

A South African study showed 29% of children dying from the respiratory disease had PJP and 22% had cytomegalovirus (CMV) on post‐mortem examination [17]. Methods to diagnose these two pathogens often rely on nasopharyngeal aspirate sampling or bronchoalveolar lavage that are not routinely performed in resource‐limited countries and do not allow for rapid diagnosis and treatment initiation [18].

Recently, recombinant synthetic antigens for *Pneumocystis jirovecii* have been used to develop a gold nanoparticle lateral flow antibody test [20]. Pooled serum from patients with PJP was used to show that this method could detect IgM against *P. jirovecii*. Further optimization and testing in different populations are needed to validate this test in CLHIV.

In addition, CMV co‐infection may play an important role in severe pneumonia and mortality [17, 21, 22]. In a study done in Kenya, CMV viremia was found in the majority of ART‐naïve children diagnosed with HIV at hospital admission, and levels >1000 IU/ml were associated with worse clinical outcomes, including longer duration of hospitalization and a higher risk of continued hospitalization or death at 15 days [23]. In this study, CMV diagnosis and quantitative measurement was done on stored samples and is not routine. Access to point‐of‐care CMV testing may allow for treatment and improved outcomes in symptomatic CLHIV admitted to the hospital with severe pneumonia.

The current WHO recommendation is to treat all infants with HIV (<1 year) with severe pneumonia empirically with cotrimoxazole, ampicillin and gentamicin due to the high prevalence of PJP in this age group [23]. For children between 1 and 5 years, the recommendation is not to use cotrimoxazole as PJP is less prevalent in this age group [23]. For children >5 years and adolescents, ampicillin and gentamicin should be used and cotrimoxazole can be added if there is a clinical suspicion of PJP.

The early use of corticosteroids in infants with a clinical diagnosis of PJP in addition to cotrimoxazole therapy significantly reduced in‐hospital mortality, and mortality at 6 months after discharge in a study from Malawi [24].

Access to valganciclovir to treat CMV remains poor in many countries. In addition, valganciclovir may cause severe side effects, such as neutropenia. Less toxic, oral treatment modalities exist (letermovir and marabavir) but are currently unavailable in most settings and have only been licensed for use in adults.

#### 2.1.3 Severe bacterial infections

Severe bacterial infections may present with varied signs according to the age of the child. A high index of suspicion is needed and empiric treatment may be necessary as blood cultures availability is limited in many low‐ and middle‐income countries. Older studies show high rates of bacteraemia in children starting ART, especially within the first 3 months of treatment [27, 28]. A more recent study, conducted in South Africa at the time of early ART initiation in children diagnosed with HIV, showed that in children with malnutrition admitted to a tertiary hospital, 6% of admission blood cultures were positive [29]. In addition, healthcare‐associated infections were predominantly Gram‐negative (39/43), and 39.5% of these were extended‐spectrum β‐lactamase positive [29].

HIV is known to be a risk factor for the development of drug‐resistant bacterial infections and there is growing evidence that HIV may increase the carriage rates of resistant bacteria in the gastrointestinal tract [32].

Two studies of hospitalized paediatric patients with bloodstream infections found that HIV infection was associated with an increased likelihood of having a resistant isolate and a corresponding increase in sepsis‐related mortality [32, 33]. Similarly, a study of paediatric bacteraemia in Tanzania found that CLHIV were more likely to receive inappropriate initial antimicrobial therapy due to drug resistance and subsequently had a higher risk of mortality compared with their HIV‐negative counterparts [34].

The lack of microbiology services and surveillance limits our knowledge of the aetiology of bacterial infections (and antibiotic resistance), making it difficult to optimize empiric treatment.

Given the increasing rates of antimicrobial resistance (AMR) in CLHIV, a comprehensive approach to AHD should consider improved AMR surveillance, improved access to treatments for resistant infections and strengthened microbiology capacity, including innovative resistance detection methods.

#### 2.1.4 Malnutrition and optimization of ART

SAM in CLHIV should be managed as in HIV‐negative children following the WHO guidelines on the treatment of SAM [36].

South African CLHIV with SAM were randomized to receive either early ART within 14 days of admission, or delayed ART at 14 days or later post‐admission and after initial nutritional stabilization (median time, 23 days) [35]. The results suggested that a short delay in ART initiation during early treatment of acute malnutrition resulted in improved immune recovery, led to faster viral suppression and improved anthropometric measures [35].

In another trial from Kenya, hospitalized CLHIV, half of whom had SAM, were randomized to receive ART either within 48 hours or in 7–14 days. There was no difference in mortality between treatment arms and the authors concluded that rapid treatment was safe and prompt initiation of ART was essential to reduce the very high mortality observed, with 21% of children dying during 6 months of follow‐up [2].

Rapid ART initiation (within 7 days of diagnosis) is a priority, particularly for children aged <5 years, although children who present with SAM need to be stabilized first.

#### 2.1.5 Cryptococcal meningitis

In contrast to adults, the cryptococcal disease is rare in CLHIV, especially under 5 years of age [36]. A laboratory‐based survey performed in South Africa estimated cryptococcal disease incidence at 47 per 100,000 HIV‐infected children in 2007. Of note, within two paediatric trial cohorts, no cases of the cryptococcal disease were reported in CLHIV <5 years of age [5, 37]. Screening for cryptococcal antigen and pre‐emptive therapy is, therefore, only recommended for adolescents [38].

Although cryptococcal meningitis is less common in children than in adults, we need to ensure that the results of the recent trial that informed the treatment of adult cryptococcal disease are extrapolated to children. The AMBITION trial showed that a single, high dose of liposomal amphotericin‐B in combination with oral antifungals, either fluconazole or flucytosine, was as effective as 7‐day amphotericin‐B‐based therapy (WHO recommended standard of care) in reducing deaths [40].

A slow‐release formulation of 5‐flucytosine (SR‐5FC) is being developed by the Drugs for Neglected Diseases initiative (DND*i*). If successful, it may simplify the management of cryptococcal meningitis, being administered orally twice daily instead of four times a day.

### 2.2 Prevention of AHD in CLHIV

Most Expanded Programme on Immunizations (EPI) vaccines are safe and effective for CLHIV [40]. Many CLHIV may have missed their EPI vaccines or were severely immunosuppressed at the time of vaccination. Catch‐up vaccination following immune reconstitution may help prevent severe bacterial infections. As COVID‐19 vaccinations become available globally for children, this may provide a unique opportunity for CLHIV to catch up other EPI vaccines.

While TB Preventive Therapy (TPT) drug combinations that include rifapentine and isoniazid are available for adults (3HP and 1HP regimens), this is not yet a reality for children. Recent data suggest that 1HP is safe and feasible in children and adolescents 2 years and above; however, drug–drug interactions with rifapentine and ART are still being studied in children [41].

As rifapentine is not yet available in many low‐ and middle‐income countries and paediatric studies on drug–drug interactions with dolutegravir (DTG) are still ongoing, isoniazid remains a valuable drug to prevent TB disease.

Cotrimoxazole is still essential to prevent PJP in CLHIV and may also prevent other infections, such as malaria and other bacterial infections. As all CLHIV less than 5 require cotrimoxazole when starting ART, a combination tablet of cotrimoxazole, isoniazid and vitamin B6 would be a convenient and practical way to prevent TB, PJP and other infections. This formulation has been used in the “REALITY” trial and has been prioritized in the PADO 4 list of treatments to address AHD in children [42].

### 2.3 Logistical and implementation issues

While efforts are ongoing to effectively scale up the AHD package of care for adults presenting with AHD, a lot remains to be done for children and adolescents.

At a national level, specific recommendations relating to AHD in children should be adopted in national guidelines. Alignment of recommendations across disease‐specific programmes is essential. For example, TPT guidelines for CLHIV should not differ between TB programmes and HIV programmes and should be available at both HIV and TB sites.

At the facility level, centres introducing the AHD package for children should aim to provide a child‐friendly environment and ensure access to child‐specific resources, such as a mid‐upper arm circumference tape, stadiometer and appropriate scales. Healthcare providers should be trained on child‐specific issues, such as growth monitoring, developmental milestones and other routine child health interventions, such as deworming and vaccination.

Procurement and supply of key commodities, such as medications and appropriate testing kits, continue to be a challenge on the ground, and estimates of paediatric‐specific needs are needed to ensure an adequate supply chain.

Monitoring and evaluation of services provided to children with AHD should be an integral part of HIV programmes. AHD‐specific registers with child‐specific indicators may help improve programmes that offer these interventions. Disaggregation of data and costing of the paediatric package of care for AHD is necessary to identify appropriate programme strengthening interventions and for appropriate drug supply.

### 2.4 Further research and evidence gaps

Multiple knowledge gaps exist in addressing the care of children with AHD.

Future research should focus on new or improved components in the package of care and identify the optimal way to deliver the package in its various components depending on the context.

**Figure 1:**
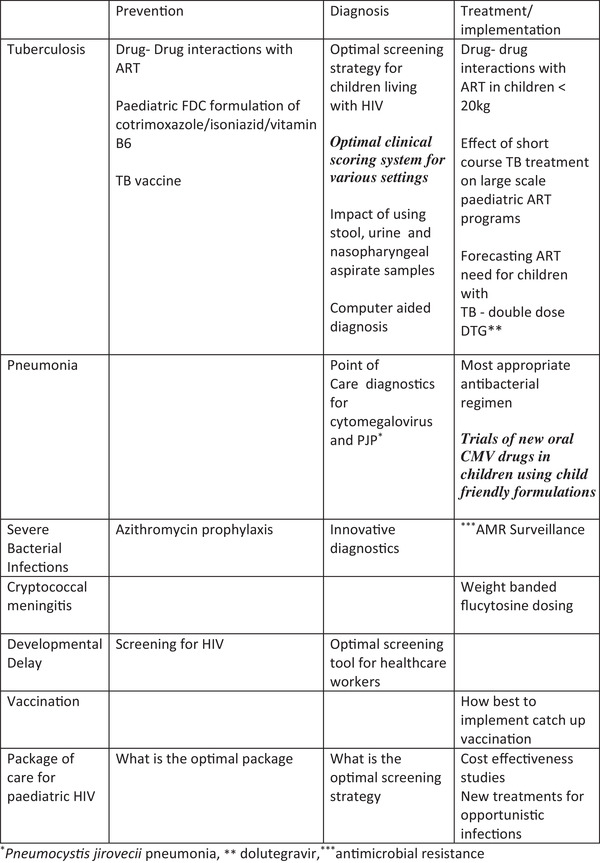
Further research and evidence gaps. ^*^ *Pneumocystis jirovecii* pneumonia, ** dolutegravir,^***^antimicrobial resistance.

Examples of this include the need for point‐of‐care diagnostics for pneumonia that include TB, PJP and CMV as well as access to innovative methods of bacterial culture for severe bacterial infections. The ongoing EMPIRICAL trial in Ivory Coast, Malawi, Mozambique, Uganda, Zambia and Zimbabwe (NCT03915366) is expected to report whether empirical treatment for TB and/or CMV in CLHIV who present with severe pneumonia is appropriate (Figure 1).

## 3 CONCLUSIONS

Children continue to die as a result of AHD. Several evidence‐based interventions have been shown to prevent and treat AHD and they have been grouped together as a package of care for ease of use; however, the optimal package of care for CLHIV remains unknown, and implementing the existing package can be challenging in some settings.

New diagnostic and therapeutic components are necessary to strengthen the package as well as innovative ways to deliver the various components. Appropriate investments in research and additional evidence generation will be critical.

All those involved in the care of children and adolescents with AHD should strive to provide holistic care that goes beyond a simple choice of ART regimen.

## COMPETING INTERESTS

All authors declare no competing interests.

## AUTHORS’ CONTRIBUTIONS

LJF and MP conceptualized the manuscript. LJF wrote the first draft. All other authors reviewed the manuscript and gave input.

All authors reviewed the final version.
